# Effectiveness of long-acting monoclonal antibodies against laboratory-confirmed RSV in children aged < 24 months and hospitalised for severe acute respiratory infection, European pilot study, 2024 to 2025

**DOI:** 10.2807/1560-7917.ES.2025.30.45.2500816

**Published:** 2025-11-13

**Authors:** Camelia Savulescu, Iris Ganser, Nathalie Nicolay, Adrien Lajot, Sandra Campos, Iván Martínez-Baz, Ana Paula Rodrigues, Mathil Vandromme, Marta Cara-Rodríguez, Aitziber Echeverría, Vânia Gaio, Marie-Pierre Parsy, Ana Roldan Garrido, Jesús Castilla, Raquel Guiomar, Sabrina Bacci, Angela MC Rose, VEBIS hospital network RSV IE group, VEBIS hospital network RSV IE group, Eva Bernaert, Reinout Naesens, Bénédicte Lissoir, Catherine Sion, Sandra Koenig, Xavier Holemans, Bénédicte Delaere, David Tuerlinckx, Marc Bourgeois, Marijke Reynders, Vanessa Verbeke, Catherine Quoidbach, Francesco Genderini, Gabriella Kollar, Nicolas Dauby, Arthur Eggerickx, Arne Witdouck, Deborah De Geyter, Eveline Van Honacker, Lucie Seyler, Siel Daelemans, Koen Magerman, Marlies Blommen, Natasja Detillieu, Veerle Penders, Melanie Delvallee, Pierre Struyven, Isabel Leroux-Roels, Pascal De Waegemaeker, Silke Ternest, Anna Parys, François Dufrasne, Sarah Denayer, Claire Brugerolles, Laurane De Mot, Peace Mpakaniye, Sébastien Fierens, Sven Hanoteaux, Yinthe Dockx, Yves Lafort, Nathalie Bossuyt, Marcos Lozano Alvarez, María Iglesias-Caballero, Gloria Pérez Gimeno, Noa Batalla Rebollo, Irene Pedrosa Corral, Manuel García Cenoz, Guillermo Ezpeleta, Nerea Egüés, Noelia Vera-Punzano, Ana Navascués, Leticia Armendáriz, Ausenda Machado, Miguel Lança, Camila Henriques, Licínia Gomes, Diogo Marques, Madelyn Rojas, Anthony Nardone

**Affiliations:** 1 Epidemiology department, Epiconcept, Paris, France; 2 European Centre for Disease Prevention and Control (ECDC), Stockholm, Sweden; 3 Epidemiology of Infectious Diseases, Sciensano, Brussels, Belgium; 4 National School of Health - Institute of Health Carlos III, Madrid, Spain; 5 National Distance Education University (UNED), Madrid, Spain; 6 Instituto de Salud Pública de Navarra – IdiSNA, Pamplona, Spain; 7 CIBER Epidemiología y Salud Pública, Madrid, Spain; 8 Epidemiology Department, National Institute of Health Doutor Ricardo Jorge, Lisbon, Portugal; 9 Complejo Hospitalario Universitario de Cáceres, Cáceres, Spain; 10 Centre Hospitalier de Wallonie Picarde, Tournai, Belgium; 11 Servicio de Vigilancia y Salud Laboral, Dirección General de Salud Pública y Ordenación Farmacéutica, Consejería de Sanidad, Presidencia y Emergencias, Andalucía, Seville, Spain; 12 National Reference Laboratory for Influenza and other Respiratory Virus, National Institute of Health Doutor Ricardo Jorge, Lisbon, Portugal; 13 The members of the VEBIS hospital network RSV IE group are listed under Collaborators.

**Keywords:** monoclonal antibodies, nirsevimab, effectiveness, RSV, Europe

## Abstract

We measured effectiveness of nirsevimab against laboratory-confirmed respiratory syncytial virus (RSV) infection in a test-negative case-control study among children aged < 24 months hospitalised for severe acute respiratory infection in three European countries. The overall effectiveness in the 2024/25 season among 2,201 children was 79% (95% CI: 58 to 89) and 85%, 78% and 69% at < 30, 30–89 and 90–215 days since immunisation. Immunisation was effective for preventing RSV-related hospitalisation in children, but effectiveness by time since immunisation needs monitoring in future seasons.

Passive immunisation with long-acting monoclonal antibodies (nirsevimab) targeting the two antigenic subgroups A and B of respiratory syncytial virus (RSV) was authorised by the European Medicines Agency for use in the European Union on 31 October 2022 [1]. Nirsevimab is recommended for preventing lower respiratory tract infection caused by RSV in infants (aged < 12 months) in their first RSV season and in toddlers (aged < 24 months) vulnerable to severe RSV during the second season [2].

The Vaccine Effectiveness, Burden and Impact Studies (VEBIS) hospital network, set up in 2021 to measure effectiveness of influenza and COVID-19 vaccines in the hospital setting, included an additional objective to measure the effectiveness of nirsevimab against laboratory-confirmed RSV among children hospitalised for severe acute respiratory infection (SARI) during the 2024/25 season. The specific objectives of the study were to measure (i) the overall immunisation effectiveness (IE) with long-acting monoclonal antibodies in eligible children aged < 24 months by age group, and (ii) RSV IE by time since immunisation.

## Immunisation effectiveness analysis

**Figure 1:**
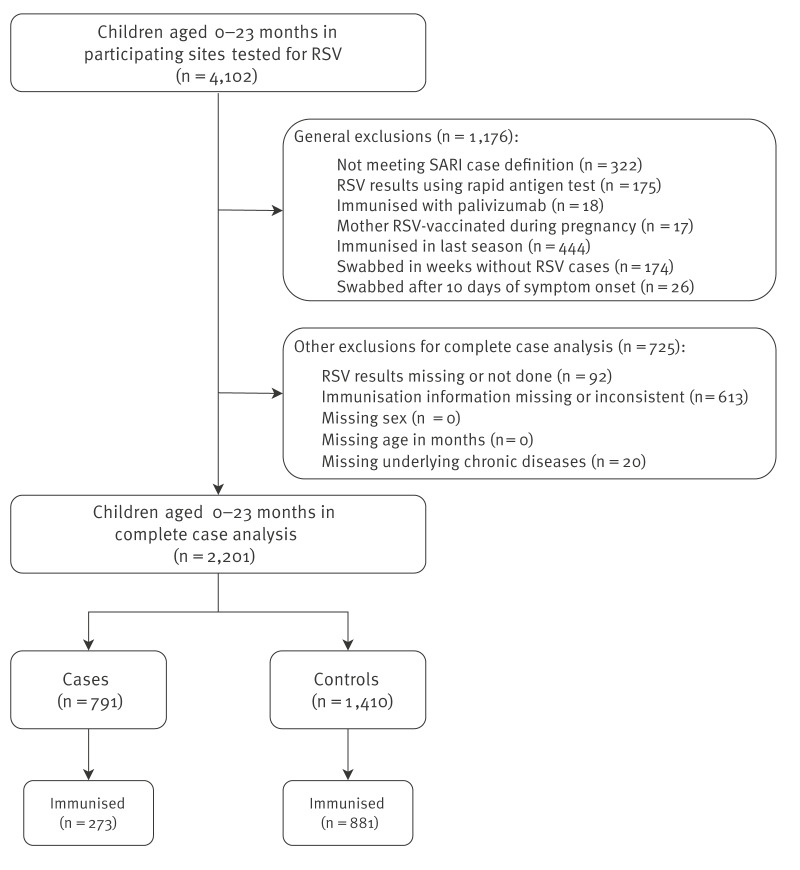
Inclusion and exclusion criteria for the VEBIS RSV immunisation effectiveness multicentre hospital study, Europe, 2024/25 season (n = 4,102)

We conducted a test-negative case-control pilot study in three countries (Belgium, Portugal and Spain). We compared the immunisation status of cases (children eligible for immunisation aged < 24 months and hospitalised for SARI [3] with a positive PCR test for RSV) and controls (eligible children hospitalised for SARI who tested PCR-negative for RSV). Immunisation eligibility was assessed according to the country-specific recommendations for immunisation [4-6]. Children were considered immunised if they received nirsevimab between September 2024 and May 2025 before testing, regardless of the dose, or their age and weight at the time of immunisation. Children immunised before September 2024 or immunised with palivizumab or through maternal vaccination were excluded from analysis. We first applied general exclusions: children who did not meet the SARI case definition, those who underwent rapid diagnostic testing only, who had symptom onset > 10 days before testing, or who were tested during weeks without RSV circulation (where RSV circulation was defined as the week of the first case to the week of the last case included in datasets from participating countries). Subsequently, additional exclusions were applied for children missing key data (case or immunisation status, age, sex, or presence of high-risk conditions) for a complete case analysis (Figure 1). In sensitivity analyses, we included children not meeting the SARI case definition (i.e. all RSV-tested by PCR), and imputed children with missing immunisation status as either immunised or not immunised.

IE: immunisation effectiveness, RSV: respiratory syncytial virus, SARI: severe acute respiratory infection, VEBIS: Vaccine Effectiveness, Burden and Impact Studies.

Using logistic regression, we calculated effectiveness as:

$IE=\left(1-odds\;ratio\;of\;immunisation\;among\;cases\;and\;controls\right)\times 100$

adjusting for age group (0–6 vs 7–23 months), sex, presence of underlying conditions, and date of testing (modelled as a spline with four internal knots, with model selection based on Akaike information criteria). We used a two-stage approach, calculating IE at the country level and then pooling site-specific IE estimates in a random-effects meta-analysis, and reported heterogeneity as τ^2^. For the time since immunisation analysis, we used the delay in days from the date of immunisation to symptom onset date grouped into three categories (< 30, 30–89, 90–215 days). Due to small sample sizes, only overall but not time-stratified IE could be calculated for the 7–23 months age group. The analyses were performed in R, version 4.4.2 (https://www.r-project.org).

## Descriptive and immunisation effectiveness results

Between September 2024 and May 2025, 4,102 hospitalised children aged < 24 months were screened, and after exclusions, we included 791 cases and 1,410 controls in our analysis (Figure 1).

**Figure 2:**
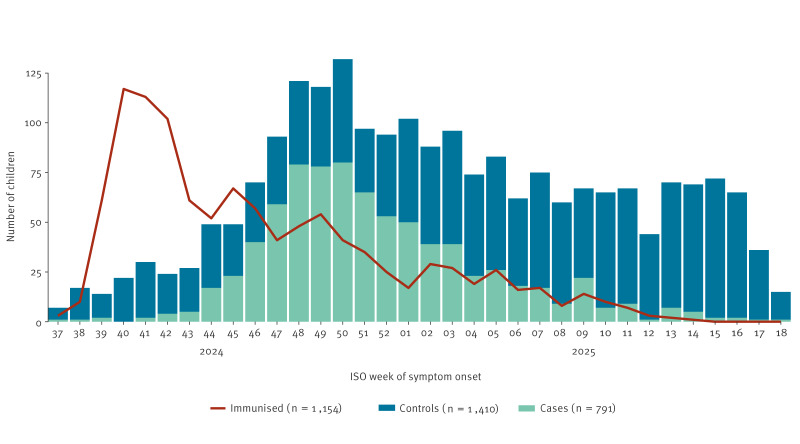
Distribution of hospitalised RSV cases and controls, by date of symptom onset, and distribution of included immunised patients, by week of immunisation, VEBIS immunisation effectiveness multicentre hospital study, Europe, 2024/25 season (n = 2,201)

ISO: International Organization for Standardization; RSV: respiratory syncytial virus; VEBIS: Vaccine Effectiveness, Burden and Impact studies.

The first RSV cases were detected in week 37/2024, with a peak observed in November and December 2024 (Figure 2). During the peak weeks, more cases than controls were recruited, while controls were more frequently enrolled at the beginning and end of the season.

Cases were more likely to present with shortness of breath (p = 0.001). Neither presence of underlying condition (p = 0.28) nor admission to intensive care (p = 0.34) was statistically significantly different between cases and controls. The median time from immunisation to symptom onset was similar between cases and controls, but longer among children aged 7–23 months than among those aged 0–6 months (Table).

**Table:** Main characteristics of RSV cases and controls, VEBIS immunisation effectiveness multicentre hospital study, Europe, 2024/25 season (n = 2,201)

Characteristic	Cases (n = 791)		Controls (n = 1,410)	
	n	%	n	%
Sex				
Female	337	42.6	594	42.1
Age (months)				
0–6	470	59.4	921	65.3
7–23	321	40.6	489	34.7
Underlying conditions^a, b^				
At least one	77	9.7	206	14.6
Country				
Country A	373	47.2	619	43.9
Country B	321	40.6	662	47.0
Country C	97	12.3	129	9.1
Symptoms^c^				
Shortness of breath	248	31.4	263	18.7
Cough	607	76.7	1,090	77.3
Fever	510	64.5	978	69.3
Not available^d^	97	12.3	129	9.1
Severity^e^				
Intensive care unit admission	90	11.4	104	7.4
Death	0	0.0	2	0.1
Length of hospital stay (days)				
Median (IQR)	3 (2–5)		3 (2–5)	
Immunised against RSV				
Yes	273	34.5	881	62.5
Median time since immunisation (days) by age group				
0–6 months (IQR)	56 (34–83)		51 (27–85)	
7–23 months (IQR)	92 (64–118)		147 (95–173)	
All < 24 months (IQR)	59 (35–89)		60 (32–111)	

IQR: interquartile range; RSV: respiratory syncytial virus; VEBIS: Vaccine Effectiveness, Burden and Impact studies.

^a^ Asthma, lung disease, diabetes, heart disease, immunocompromising conditions, liver disease, neuromuscular conditions, renal disease.

^b^ Presence of an underlying condition was similar between cases and controls (p = 0.28).

^c^ Cases were more likely to present with shortness of breath (p = 0.001).

^d^ No information on symptoms was available for children from Country C.

^e^ Admission to intensive care was similar between cases and controls (p = 0.34).

**Figure 3:**
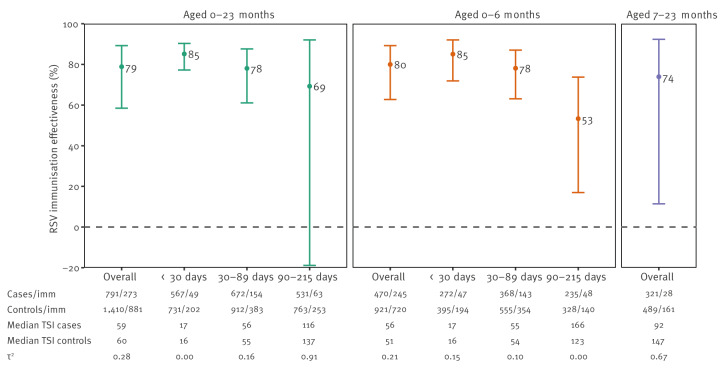
Immunisation effectiveness against RSV in children aged < 24 months, by age group and time since immunisation, 2-stage pooled analysis, VEBIS multicentre hospital study, Europe, 2024/25 season^a, b^

Pooled overall IE was 79% (95% confidence interval (CI): 58 to 89, τ^2^ = 0.28) (Figure 3). The IE declined from 85% (95% CI: 77 to 90) at < 30 days from immunisation to 78% (95% CI: 61 to 88) at 30–89 days from immunisation and 69% (95% CI: −19 to 92) at 90–215 days from immunisation. Among infants aged 0–6 months, the overall IE was 80% (95% CI: 63 to 89) and was 85% (95% CI: 72 to 92) at < 30 days from immunisation, 78% (95% CI: 63 to 87) at 30–89 days, and 53% (95% CI: 17 to 74) at 90–215 days from immunisation. In children aged 7–23 months, overall IE was 74% (95% CI: 11 to 92).

imm: immunised; RSV: respiratory syncytial virus; τ^2^: heterogeneity; TSI: time since immunisation; VEBIS: Vaccine Effectiveness, Burden and Impact studies.

^a^ Adjusted by age, sex, country, at least one underlying condition, and time of onset.

^b^ Due to sparse data, we were not able to stratify by time since immunisation among children aged 7–23 months.

Sensitivity analyses gave similar results. Including all RSV-tested infants gave an IE of 79% (95% CI: 59 to 89). Imputing missing immunisation status as immunised gave an IE of 64% (95% CI: 55 to 71); including them as non-immunised: 75% (95% CI: 59 to 85), closer to the complete-case analysis results. In the Supplementary material we show IE results in forest plots for each country and combined, overall and by age group and by time since immunisation.

## Discussion

Respiratory syncytial virus is a highly contagious RNA virus [7] infecting almost all children at least once before the age of 2 years [8] and with a clear seasonality of infection from late autumn to early spring in Europe [9]. Reinfections are common due to short-lived or incomplete protection from previous infections [10]. The infection in young children is often severe, leading to hospitalisation [11], but it can be prevented by using long-acting monoclonal antibodies. In this pilot study, we estimated the effectiveness of nirsevimab in young children from three European countries belonging to the VEBIS hospital network. Our results indicate a high IE overall and by age group, although the IE among children aged 7–23 months was less precise than in those aged 0–6 months. Results by time since immunisation overall and in infants aged 0–6 months suggest that protection declines after 90 days post immunisation. The low number of children immunised in the 90–215 days category for time since immunisation in two of the three sites, and high observed heterogeneity, precluded more in-depth pooled analysis in this stratum.

Our results overall and in the age group 0–6 months are in line with efficacy data from clinical trials [12], observational studies from the 2023/24 season [13,14] and for the current season from the United States (US) (Adam MacNeil, personal communication, ACIP meeting, June 2025: https://www.cdc.gov/acip/downloads/slides-2025-06-25-26/03-MacNeil-Mat-Peds-RSV-508.pdf) and Italy [15]. However, we noted a lower IE point estimate ≥ 90 days after immunisation, similar to the results of a US study from the previous season [16] and in line with the reported terminal half-life of nirsevimab (e.g. ca 71 days for a 5 kg infant and decreasing with infant weight [17]), while the phase 3b Harmonie study [18] reported a high efficacy for up to 180 days. The difference may be related to the way the analysis was performed; while we stratified by time since immunisation, the Harmonie study pooled data from all time points. If validated in future seasons, our results by time since immunisation underscore the critical role of RSV surveillance for optimising the start of immunisation programme with long-acting monoclonal antibodies, rather than relying on programmatic schedules. This will be particularly important during delayed RSV seasons.

The main limitation of this pilot study is the amount of missing data (15%) on immunisation status. However, the overall proportion immunised among controls aligned with national estimates, and sensitivity analyses indicated that missing immunisations had limited impact. Secondly, we noted heterogeneity between sites mainly related to immunisation recommendations and the use of SARI case definition. Efforts will be made to reduce heterogeneity by ensuring the implementation of a common protocol in the next season. Thirdly, reduced specificity of the SARI case definition in young children may have led to an underestimated IE, as less severe RSV cases might have been included. Lastly, important additional information, such as previous RSV infection, gestational age, birthweight, co-infections, and dose and timing of immunisations, was not collected in this pilot season.

## Conclusion

High long-acting monoclonal antibody IE (79%) was confirmed for the 2024/25 season. A core European Union 2025–26 protocol has been developed based on lessons learned from this pilot study to improve data collection, increase the sample size at the site level and extend the study to more sites for more precise estimates by time since immunisation. Real-world studies remain important to address key public health questions, such as whether early-immunised infants remain protected throughout the entire RSV season, the IE by RSV type, and among older high-risk children.

## Data Availability

Data are available from the corresponding author upon reasonable request.

## Supplementary Data

1446000 Supplementary material
