# Supplementary material for: Effectiveness of long-acting monoclonal antibodies against laboratory-confirmed RSV in children aged < 24 months and hospitalised for severe acute respiratory infection, European pilot study, 2024 to 2025
Source: Euro Surveill. 2025 Nov 13;30(45):2500816. doi: 10.2807/1560-7917.ES.2025.30.45.2500816 (PMC12633706; doi:10.2807/1560-7917.ES.2025.30.45.2500816)
Supplement: Supplementary material [file 25-00816_SAVULESCU_Supplement.pdf]

This supplementary material is hosted by Eurosurveillance as supporting information alongside the article “Effectiveness of long-acting monoclonal antibodies against laboratory-confirmed RSV in eligible children <24 months hospitalised for severe respiratory acute infection, European pilot study 2024–2025”, on behalf of the authors, who remain responsible for the accuracy and appropriateness of the content. The same standards for ethics, copyright, attributions and permissions as for the article apply. Supplements are not edited by Eurosurveillance and the journal is not responsible for the maintenance of any links or email addresses provided therein.

## Forest plot of RSV IE, Aged 0-23 months, Overall

| Country | imm.cases | imm.controls | TSI cases (IQR) | TSI controls (IQR) | IE (%) | $\tau^2$ |
|---|---|---|---|---|---|---|
| Country A | 47/369 | 262/603 | 39 (26-54) | 60 (37-94) | 86 (78-91) | |
| Country B | 175/321 | 486/655 | 72 (40-108) | 63 (32-125) | 61 (44-73) | |
| Country C | 47/97 | 110/129 | 49 (34-61) | 42 (19-78) | 84 (67-93) | |
| **Combined** | **273/791** | **881/1410** | **59 (35-89)** | **60 (32-111)** | **79 (58-89)** | **0.28** |

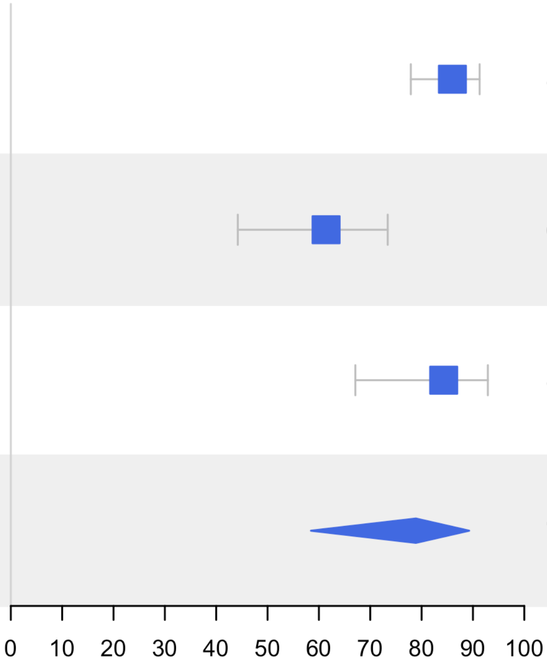

## Forest plot of RSV IE, Aged 0-23 months, <30 days

| Country | imm.cases | imm.controls | TSI cases (IQR) | TSI controls (IQR) | IE (%) | $\tau^2$ |
|---|---|---|---|---|---|---|
| Country A | 16/338 | 47/388 | 18 (12-26) | 16 (12-24) | 81 (63-91) | |
| Country B | 26/172 | 117/286 | 16 (12-21) | 17 (11-23) | 84 (70-91) | |
| Country C | 7/57 | 38/57 | 16 (14-26) | 14 (11-19) | 94 (83-98) | |
| **Combined** | **49/567** | **202/731** | **17 (13-24)** | **16 (11-23)** | **85 (77-90)** | **0** |

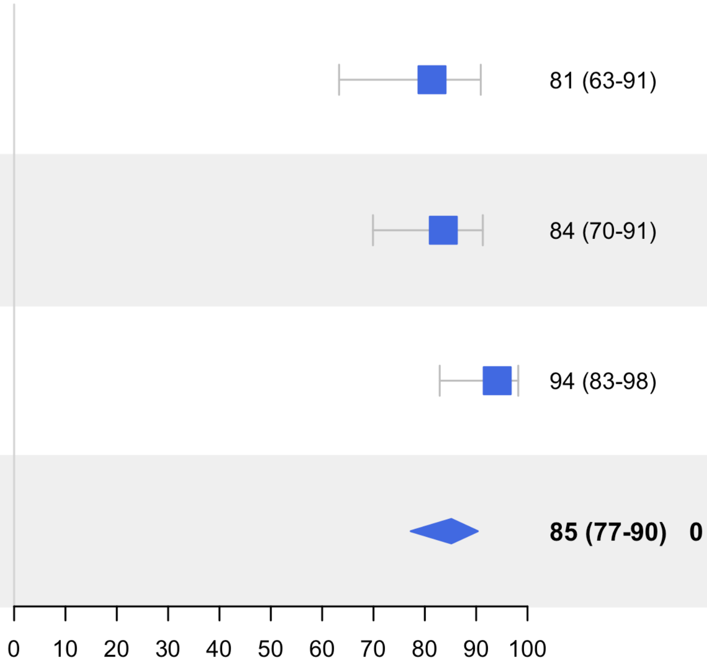

## Forest plot of RSV IE, Aged 0-23 months, 30-89 days

| Country | imm.cases | imm.controls | TSI cases (IQR) | TSI controls (IQR) | IE (%) | $\tau^2$ |
|---|---|---|---|---|---|---|
| Country A | 30/352 | 144/485 | 50 (39-61) | 56 (43-70) | 87 (78-93) | |
| Country B | 87/233 | 187/356 | 64 (44-76) | 54 (42-72) | 71 (53-83) | |
| Country C | 37/87 | 52/71 | 51 (37-61) | 52 (38-74) | 69 (28-87) | |
| **Combined** | **154/672** | **383/912** | **56 (40-70)** | **55 (42-71)** | **78 (61-88)** | **0.16** |

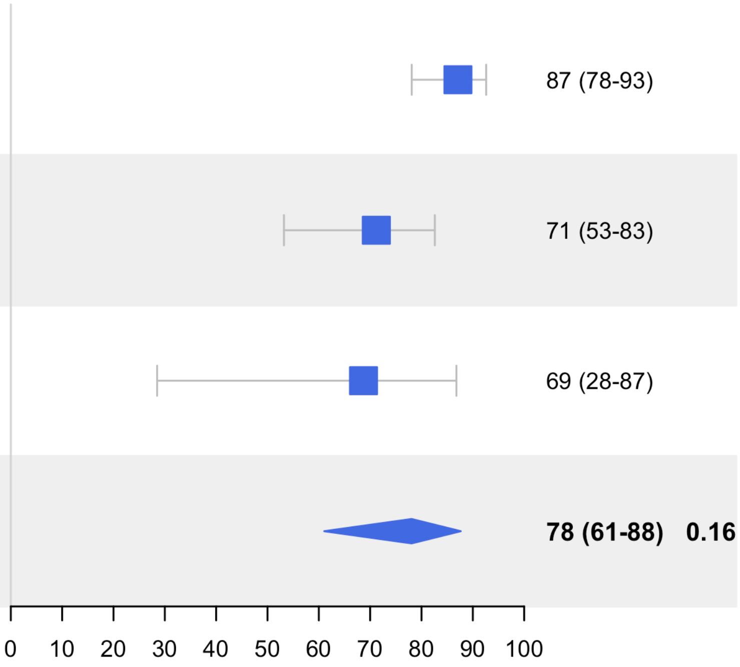

## Forest plot of RSV IE, Aged 0-23 months, ≥90 days

| Country | imm.cases | imm.controls | TSI cases (IQR) | TSI controls (IQR) | IE (%) | $\tau^2$ |
|---|---|---|---|---|---|---|
| Country A | 1/323 | 71/412 | 91 (91-91) | 137 (110-156) | 92 (60-100) | |
| Country B | 62/208 | 182/351 | 118 (103-132) | 142 (118-172) | 26 (-23-55) | |
| Country C | 3/53 | 20/39 | 101 (99-125) | 115 (107-123) | 79 (-17-97) | |
| **Combined** | **63/531** | **253/763** | **116 (101-132)** | **137 (115-166)** | **69 (-19-92)** | **0.91** |

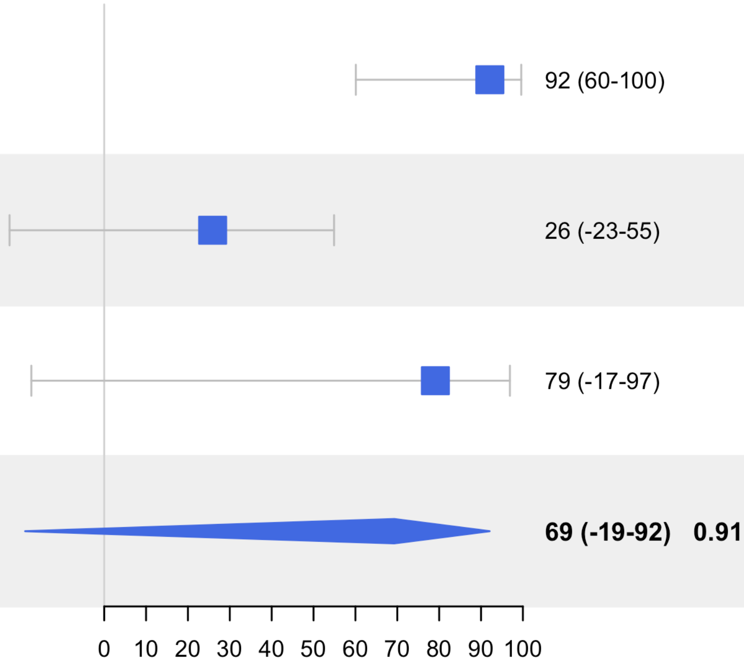

## Forest plot of RSV IE, Aged 0-6 months, Overall

| Country | imm./cases | imm./controls | TSI cases (IQR) | TSI controls (IQR) | IE (%) | $\tau^2$ |
|---|---|---|---|---|---|---|
| Country A | 44/176 | 216/340 | 39 (26-56) | 53 (32-79) | 86 (76-92) | |
| Country B | 153/208 | 384/448 | 68 (38-101) | 54 (28-95) | 65 (45-78) | |
| Country C | 44/82 | 101/114 | 48 (34-61) | 38 (16-74) | 86 (68-94) | |
| **Combined** | **245/470** | **720/921** | **56 (34-83)** | **51 (27-85)** | **80 (63-89)** | **0.21** |

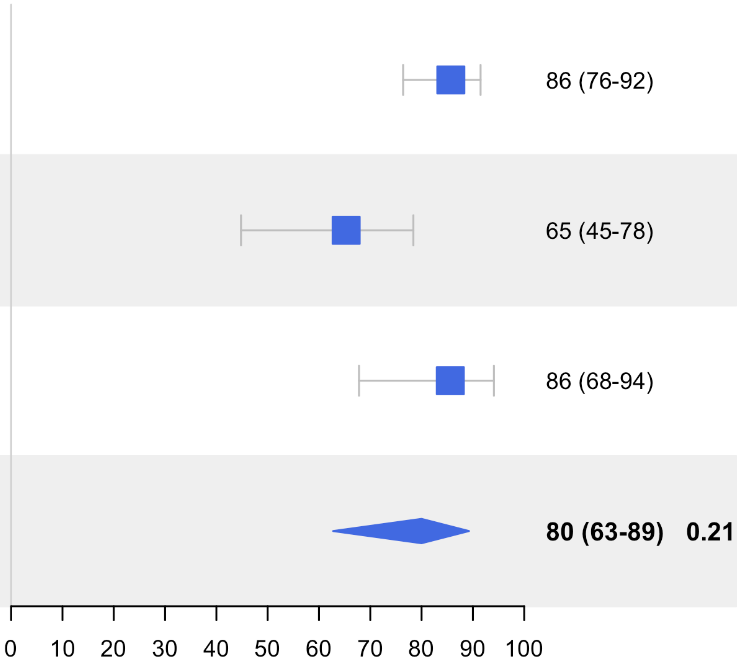

## Forest plot of RSV IE, Aged 0-6 months, <30 days

| Country | imm./cases | imm./controls | TSI cases (IQR) | TSI controls (IQR) | IE (%) | $\tau^2$ |
|---|---|---|---|---|---|---|
| Country A | 14/146 | 47/171 | 18 (10-24) | 16 (12-24) | 84 (68-92) | |
| Country B | 26/81 | 110/174 | 16 (12-21) | 18 (11-23) | 78 (60-88) | |
| Country C | 7/45 | 37/50 | 16 (14-26) | 13 (11-19) | 94 (82-98) | |
| **Combined** | **47/272** | **194/395** | **17 (12-24)** | **16 (11-23)** | **85 (72-92)** | **0.15** |

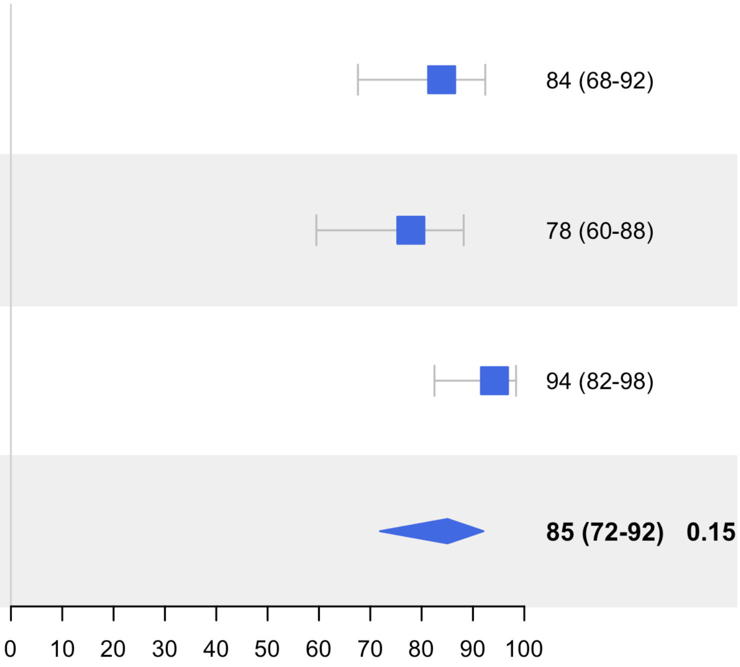

**Forest plot of RSV IE, Aged 0-6 months, 30-89 days**

| Country | imm./cases | imm./controls | TSI cases (IQR) | TSI controls (IQR) | IE (%) | $\tau^2$ |
|---|---|---|---|---|---|---|
| Country A | 29/161 | 133/257 | 51 (39-61) | 56 (42-70) | 86 (75-92) | |
| Country B | 80/135 | 170/234 | 62 (42-74) | 54 (41-71) | 69 (46-83) | |
| Country C | 34/72 | 51/64 | 50 (37-61) | 52 (38-73) | 76 (40-91) | |
| **Combined** | **143/368** | **354/555** | **55 (40-70)** | **54 (41-71)** | **78 (63-87)** | **0.1** |

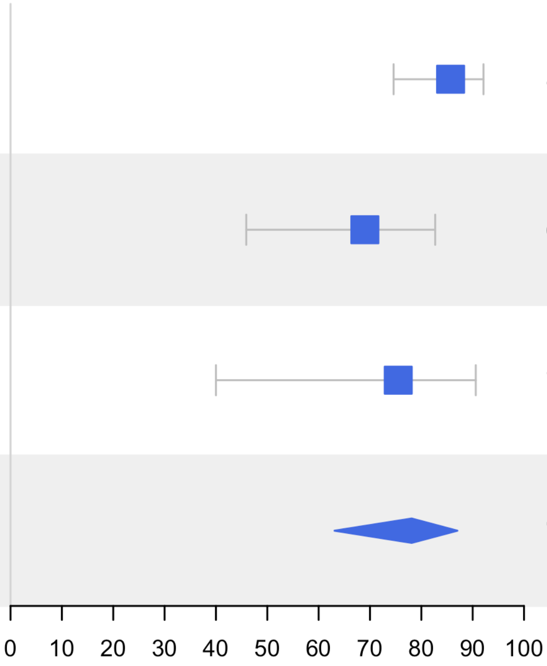

## Forest plot of RSV IE, Aged 0-6 months, ≥90 days

| Country | imm./cases | imm./controls | TSI cases (IQR) | TSI controls (IQR) | IE (%) | $\tau^2$ |
|---|---|---|---|---|---|---|
| Country A | 1/133 | 36/160 | 91 (91-91) | 124 (104-148) | 90 (44-100) | |
| Country B | 47/102 | 104/168 | 118 (104-132) | 124 (106-142) | 48 (3-72) | |
| Country C | 3/41 | 13/26 | 101 (99-125) | 116 (112-121) | 36 (-386-92) | |
| **Combined** | **48/235** | **140/328** | **116 (102-132)** | **123 (106-141)** | **53 (17-74)** | **0** |

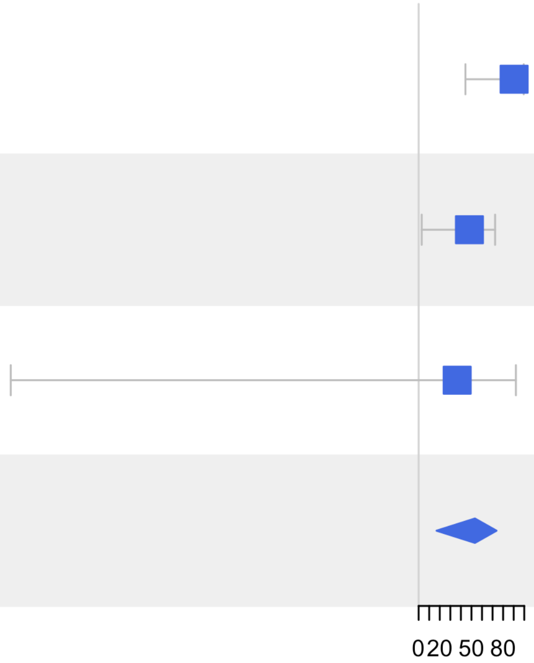

## Forest plot of immunisation effectiveness in 7-23 month-olds

| Country | imm./cases | imm./controls | TSI cases (IQR) | TSI controls (IQR) | IE (%) | $\tau^2$ |
|---|---|---|---|---|---|---|
| Country A | 3/193 | 46/263 | 28 (24-36) | 135 (92-159) | 88 (61-97) | |
| Country B | 22/113 | 102/207 | 104 (81-129) | 159 (106-179) | 44 (-9-71) | |
| Country C | 3/15 | 9/15 | 59 (46-66) | 103 (99-123) | 87 (-57-99) | |
| **Combined** | **28/321** | **161/489** | **92 (64-118)** | **147 (95-173)** | **74 (11-92)** | **0.67** |

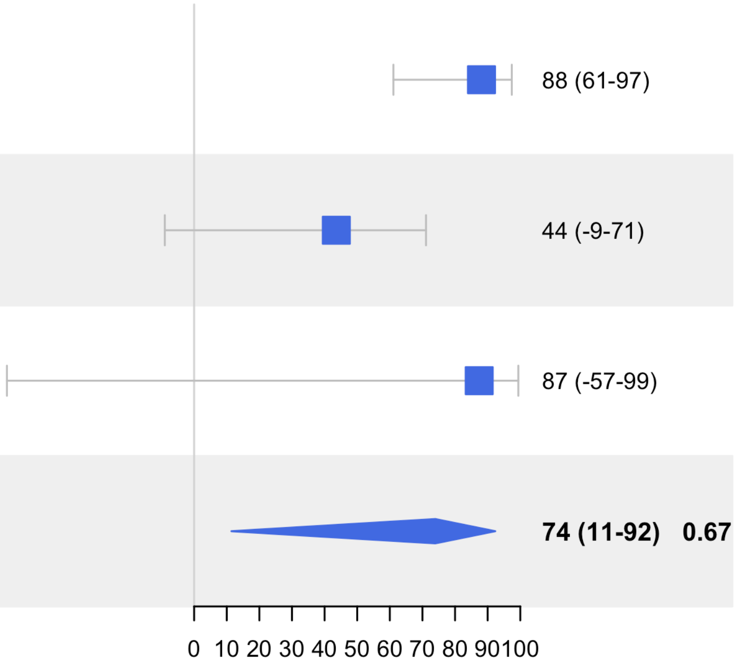


IE: immunisation effectiveness; imm: immunised; IQR: inter-quartile range; RSV: respiratory syncytial virus; TSI: time since immunisation.
